# Understanding the Farmland Rights Confirmation Policy from the Perspective of Farmers: Evidence from Jiangxi, China

**DOI:** 10.3390/ijerph191811295

**Published:** 2022-09-08

**Authors:** Lingying Lu, Guoliang Xu, Zhiyuan Li, Chunyan Wan

**Affiliations:** 1 School of Tourism and Urban Management, Jiangxi University of Finance and Economics, Nanchang 330032, China; 2 Institute of Ecological Civilization, Jiangxi University of Finance and Economics, Nanchang 330013, China

**Keywords:** farmland rights confirmation policy, farmers’ behavior, farmland transfer, mediation effect, China

## Abstract

Since the 1970s, although the Chinese central government has constructed farmland rights confirmation policy to stimulate the vitality of rural land elements, it is rare to discuss the effectiveness of the policy from the perspective of farmers. This paper applies the deep interview and questionnaire to present an analysis framework consisting of “policy implementation–input behavior in agricultural production–farmland transfer” and testifies the framework with the mediation model. The findings show that, in general, the farmland rights confirmation policy (FRCP) has a significant negative impact on leasing out farmland and a significant positive impact on leasing in farmland. In particular, for farmland leasing out, the mediating effects of agricultural capital input and agricultural time input account for 15.504% and 14.536% of the total effect, respectively. In addition, for leasing farmland in, these two mediating effects accordingly account for 13.798% and 12.155% of the total effect. It is worth noting that in the future, we should understand FRCP based on the consideration of farmers’ behavior in a given local context, while also focusing on policy implementation as well as policy design.

## 1. Introduction

It has been mutually agreed that the efficiency of agricultural land can be promoted via the implementation of appropriate policies [1,2,3,4,5,6,7]. While vigorously increasing input in agricultural production [8,9], in the context of rapid urbanization development, the issue of how farmers’ restrictions regarding agricultural land can be loosened has been an important direction in rural land system reform in China [10,11].

**Figure 1:**
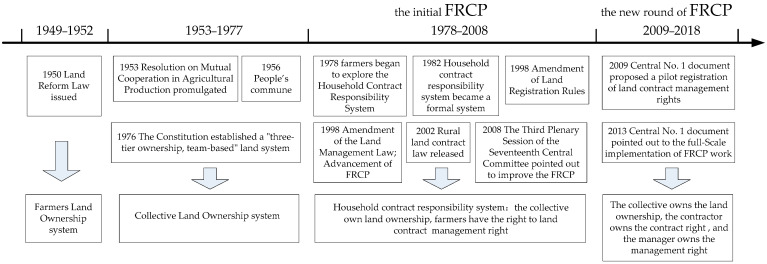
Evolutionary process of rural land rights system reform in China since 1949.

Generally, China’s rural land rights system reform has gone through different stages since 1949 [12]. This evolutionary process is divided into four stages based on the tenure relationship between farmers and rural land (Figure 1). In 1950, the Land Reform Law was promulgated and implemented, establishing a socialist land system with “farmers ownership” as the mainstay. Although farmers owned the land, there were still many limitations such as fragmented production, low technology, lack of tools, and so on. In order to further demonstrate fairness and quickly achieve common prosperity, the rural land system of “collective ownership” was established in 1953. In 1978, in Xiaogang Village, Anhui Province, farmers began to spontaneously explore the Household Contract Responsibility System (HCRS). In the following years, HCRS was widely implemented and officially recognized by the No. 1 document of the Central Government in 1982, which pioneered the separation of land ownership and use rights in China and fully liberated and developed the rural productive forces. Subsequently, China entered the FRCP period, which consisted of an initial phase (1978–2008) and a new round phase (2009–2018). Between 1978 and 2008, China’s land system reform began to be oriented towards efficiency as a priority. In order to improve the efficiency of agricultural production, the Chinese government started the initial exploration of FRCP. However, these problems emerged in the initial phase of FRCP, for example, frequent farmland modification, ambiguous property rights definitions, and a lack of land contractual management certificates [13,14,15,16]. In particular, the rapid urbanization that began in the 1990s accelerated the rate of rural-to-urban migration [17], thereby exacerbating these problems to some extent. As a result, in 2009, the Chinese government began a new round of FRCP pilots, which were introduced nationwide in 2013, and completed within five years.

Obviously, from the above stages, it can be seen that the tenure relationship between Chinese farmers and land has undergone an evolution from a “single right” to a “bundle of rights”. There is no doubt that the aim of FRCP was to stimulate the vitality of rural land elements [18]. According to property rights theory, the FRCP has encouraged farmers to transfer farmland by offering policy assurances and reducing the costs in the process of farmland transfer [14,19]. Conversely, there are also arguments that FRCP may have limited positive effects on farmland transfer and may even be inhibitory to some extent [20]. We agree that it is too early to draw judgment on the effectiveness of the policy, in part because we cannot ignore the complicated backdrop of FRCP in China. In terms of policy management, the FRCP has nearly achieved the policy goal of increasing the efficiency of land transfer by adopting a set of market transaction rules [14]. However, from the perspective of farmers, this problem may reveal a more complex scenario, with one reason being the vagueness of government policy [21], and another being the ambiguous role of FRCP, particularly in the setting of China’s diverse social and economic environment.

Therefore, farmers’ transaction behavior is not only economic behavior but a comprehensive behavior associated with various situations. It is necessary to understand the FRCP based on the consideration of farmers’ behavior, which is different from a policy perspective and will provide a more detailed explanation. Therefore, this paper explores the influencing mechanism of the FRCP on farmland transfer behavior. First, a conceptual framework is built that includes the characteristics “policy implementation–input behavior in agricultural production–farmland transfer”. We focus on farmers’ agricultural input behavior in agricultural production, which is divided into two aspects: agricultural capital input and agricultural time input. Second, the research analyzes the direct and indirect effects of the FRCP on farmland transfer behavior. Third, it reveals the role of farmers’ input behavior in mediating the influence of FRCP on farmland transfer behavior. The key contribution of the study is the inclusion of the mediating variable “farmers’ agricultural production input” to explain the process by which the FRCP affects farmland transfer, which objectively conveys farmers’ value assessment on the implementation of FRCP.

## 2. Literature Review

### 2.1. Farmland Rights Confirmation Policy: Theoretical and Empirical Evidence of Property Rights Economics

As part of the reform of China’s rural land system, FRCP has led to an increase in the duration, intensity, and guarantee of farmers’ land rights, which not only reduces transaction costs but also stimulate farmland transfer [19,22,23,24]. Simultaneously, the inherent worth of land resources has also grown as a result of FRCP [25]. Some scholars have proposed that that FRCP may facilitate farmland transfer under the analysis of property rights theory [18,26]. According to Cheng et al., controlling for other variables, FRCP increased the likelihood of farmers participating in farmland transfer by 4.9%, while the average amount of land transfer per household in villages increased by 246.67 m^2^, which is almost twice as much as in villages without the implementation of FRCP, and the land rental rate increased by 43.3% [18]. According to worldwide experience, the confirmation or stability of land rights has a significant influence on land circulation. Scholars in the Dominican Republic discovered that secure land tenure improved land rental market activity, resulting in a 21 percent rise in land leases and a 63 percent increase in property leased to the poor [27]. Landowners with secure property rights are more likely to engage in the land transfer market in Nicaragua than those who do not [28]. Land registration in Ethiopia might improve tenure security by securing usufruct rights over land and resolving conflicts that occur from competition for irrigable land. Other significant advantages include lessening landowners’ anxiety about losing their property to tenants [29].

Farmland transfer markets are seen as regular commodity exchange markets under the property rights theory, in particular. Property rights have an impact on the farmland transfer market through a variety of factors, including social security, employment outside of agriculture, transaction prices, transaction costs, linkage of rural factor market, and more [30,31,32,33,34]. Lin et al. [30] found that the impact of FRCP on the transfer of farmland is uncertain and its effect depends on the combined impact created by the transmission mechanism, such as agricultural production incentives, transaction cost, transaction price, and linkage of the rural factor market. Ye et al. [34] use a mediating effects model and find that FRCP encourages the transfer of farmland by increasing the value of farmland and reducing transaction costs to increase the net income from farmland transfer.

### 2.2. Farmland Rights Confirmation Policy: Theoretical and Empirical Evidence of Behavioral Economics

Due to the uniqueness of the farmland transfer market, some scholars have introduced analytical concepts such as endowment effects to explain the behavioral economic logic behind farmland transfer transactions and pointed out that the confirmation of rights does not always facilitate the behavior of farmland transfer and may even have a disincentive effect [35]. Farmers have a significant endowment effect on farmland, which causes those who lease farmland out to overestimate the value of their own land. Once it exceeds the price accepted by those who lease farmland in, it becomes more challenging for both sides to come to an agreement, which puts a damper on the farmland transfer [20]. Additionally, the endowment effect is frequently closely related to farmers’ individual views. Farmers specifically view their farms as status symbols or spiritual anchors. It is an irreplaceable and personalized asset for them. Even Nevertheless, when rights are confirmed and become more widely recognized, this subjective perception will grow [36]. Farmers specifically view their farms as status symbols or spiritual anchors. It is an irreplaceable and personalized asset for them. Even, when rights are confirmed and become more widely recognized and legitimate, this subjective perception will grow.

In fact, farmland is more of a personal emotional asset for farmers than it is a regular commodity [25]. Therefore, it must be acknowledged that farmland represents farmers’ subjective perceptions. This further demonstrates that transferring farmland is a complex transaction that reflects the identity, feelings, and perceptions of farmers as well as their rights and interests. According to the literature that is currently accessible, the institutional credibility of the land confirmation system, agricultural production inputs, and other factors all have an impact on farmers’ behavior as rational economic agents and participants in farmland transfer [25,37,38,39]. Lin et al. confirmed that FRCP stimulates farmers’ enthusiasm to engage in agricultural production and operation, and then urges them to increase farmland leasing in and reduce farmland leasing out [37]. Moreover, Li et al. found that the effect of land rights confirmation is lagging behind, and the longer the time period of confirmed right, the stronger farmers’ willingness to land transfer [38].

In contrast to the property rights theory, behavioral economics emphasizes the uniqueness of the market for farmland transfer in China. That is, the Chinese farmland transfer market is composed of a network of economic and social interactions, which is different from the economic transactions of generic components or commodities as defined by Coase. The practical impact of the FRCP remains a hot subject in China’s reform of the rural land system. The current study makes two contributions: first, it provides empirical data on whether policy influences farmland transfer behavior; and second, it analyses farmers’ agricultural production input behavior, since policy’s impact appears to be restricted by farmers’ individual decision-making behavior.

## 3. Theoretical Analysis and Hypothesis

The FRCP is a kind of national empowerment whose goal is to clearly specify who owns the contractual management right to land (use right) in rural areas [9]. Farmers have been granted clearer, fuller, and official rural land contract management rights as the FRCP has been deepened and improved [40]. On the one hand, land ownership in rural China is strictly an institutional framework, resulting in intentionally ambiguous agricultural property rights [41]. This uncertainty has served as a lubricant for institutional policy in China, which is in the midst of an economic revolution. However, as industrialization and urbanization have progressed, the flaws in this type of unclear agricultural property rights management have become more apparent. The confirmation of agricultural land rights, on the other hand, improves the property rights system, maintains the long-term stability of land contracting relations, and increases the intrinsic value of land resources through legal provisions in terms of stability, security, and guarantee of property rights. There are four reasons why the FRCP should be implemented to boost farmers’ agricultural input.

First, land certification is an effective strategy for securing tenure, boosting farmer confidence in their land rights, and increasing land-related inputs and crop yield [19]. To put it another way, the impacts of secured ownership on loan availability and input incentives indicate that farmers without secure ownership will spend less on inputs and land improvements, employ fewer variable inputs, and produce less per unit of land [8,42]. Farmers only invest in land when they expect a steady return; therefore, they prefer to invest when their expectations are predictable [43].

Second, solid farmland property rights may safeguard asset owners’ future income from being taken away by others, increase future income expectations, and lower the regret impact of agricultural input, all of which can boost farmers’ long-term capital input incentive [44]. Similarly, if land rights stay secure and constant, farmers will be more prepared to invest in long-term inputs and increase the amount of organic fertilizer used [45]. Furthermore, steady expectations strengthen farmers’ sense of connection to the land.

Third, FRCP certificates provide legal guarantees for land transactions and financial credit, as well as encourage land input [46]. The confirmation of farmland rights through legal provisions allows farmers’ land property rights or usage rights to be clearly defined and effectively guaranteed, which can have a positive impact on not only reducing landowners’ fear of land transfer but also promoting gender equality and reducing land disputes [29].

Fourth, measures that affirm rights allow farmers to obtain greater finance capital. Farmers’ contracted management rights have been defined by the FRCP, allowing them to seek loans to alleviate economic stress. In addition, their passion for output might be boosted. As a result, the prospect of increasing agricultural input grows.

Together, the foregoing reasons suggest that the FRCP may increase farmers’ passion for agricultural output and encourage them to invest. Rural families are more likely to lease farmland in and less likely to lease farmland out when farmers’ ardor for agricultural output grows. We can offer the following four hypotheses since an increase in agricultural input would raise demand for lands while decreasing supply:

**Hypothesis** **1 (H1).** *FRCP may inhibit the behavior of farmland leasing out.*

**Hypothesis** **2 (H2).** *FRCP may stimulate the behavior of farmland leasing in.*

**Hypothesis** **3 (H3).** *FRCP may inhibit the behavior of farmland leasing out through agricultural production input*.

**Hypothesis** **4 (H4).** *FRCP may stimulate the behavior of farmland leasing in through agricultural production input.*

## 4. Materials and Methods

### 4.1. Research Design and Data Collecting

This study mostly uses field research to acquire data, based on literature research. In general, we do our study in four stages.

**Figure 2:**
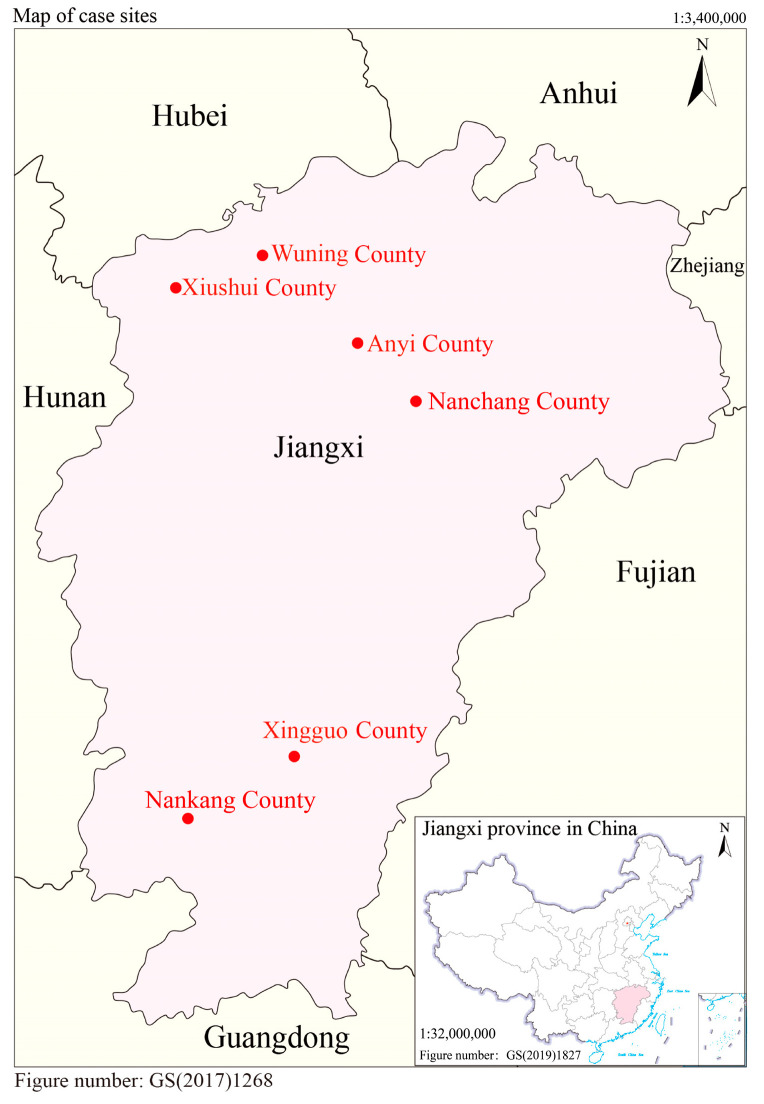
Location of the six case sites. Notes: these two maps are downloaded from http://bzdt.ch.mnr.gov.cn/ (accessed on 1 August 2022), without changing the boundaries. The figure numbers are GS(2017)1268, GS(2019)1827, respectively, the scales are 1:3,400,000, 1:32,000,000.

At the preparation stage, we chose three cities named Jiujiang, Nanchang, and Ganzhou, which are located in the northern, middle, and southern regions of Jiangxi Province, respectively, to improve the statistical representativeness of the sample (Figure 2). Following the principle of random selection, we chose Wuning and Xiushui County in Jiujiang, Nanchang, and Anyi County in Nanchang, Xingguo, and Nankang County in Ganzhou from the list of counties in the three cities.

After that, our research team focused on Nanchang County from January to February 2021, and we chose January as the month to do field research since farmers working in cities began to return home to celebrate the Spring Festival, considerably increasing the practicality of our study. During this period, we use the semi-structured in-depth interview method to primarily expose three questions. To ensure a plethora of information from interviewers, the average speaking length with each interviewee was 1.1 h, and we eventually collected 20 samples.

Our study team then created a questionnaire based on the literature analysis and preliminary research. The FRCP, agricultural production input, farmland transfer, household head characteristics, household characteristics, and contractual land characteristics were among the specific variables chosen. During the questionnaire design process, we went through dozens of rounds of changes to the content, text, format, and other aspects of the questionnaire, as well as sending out about 50 pre-test questionnaires, which were collected and then analyzed for reliability and validity to come up with the final version.

Finding a representative farmer sample to conduct a questionnaire survey is the final step of the field inquiry. To do so, we mainly conduct it with the head of the household. Our research team selected two villages in each county, with the support of the local government department. We choose the household number randomly by computer during the questionnaire survey to decide the households that need to be investigated; if there is no one in the household, we look for the next one in an ascending single-digit way. In general, 12 administrative villages were surveyed at this stage, with around 15 peasant homes in each village from February to March 2021.

Throughout the whole research, 200 questionnaires were given to the head of the household, with 22 of them being invalid due to missing information, leaving 178 valid surveys. The questionnaire had an effective response rate of 89.00%. The distribution of the sample size for each case is shown in the following table.

### 4.2. Methods

#### 4.2.1. Variable Descriptions

(1) Main Variables

Definitions and descriptions of the variables used in this study are shown in Table 1. This research experimentally evaluates and conceptually analyzes the process of FRCP-agricultural production input-farmland transfer from the standpoint of farmers’ land transfer behavior. As a result, the explained variable is cropland transfer behavior, which can be classified into two categories: whether to lease out or whether to lease in.

The core explanatory variable is FRCP. The influence of FRCP policy has a degree of hysteresis. The variable “presence of certificate” has been used in previous research to assess the impact of FRCP on agricultural circulation. The main reason for this is that the logic of system development has been applied to system operating issues; nevertheless, this may lead to bias. Therefore, the paper applies the item “how many years have passed since you obtained the certification” to represent the meaning of the core explanatory variable.

(2) Mediating and Control Variables

The mediating variable, agricultural production input, may be separated into two components, according to the literature: agricultural capital input and agricultural time input (see Table 1).

The control variables used in this paper are: (1) household head characteristics, such as age, education level, and village cadre experience of the household head; (2) family characteristics, such as nonfarmer size (i.e., the proportion of nonagricultural labor force in the family) and agricultural machinery; and (3) contracted farmland characteristics, such as contracted farmland area, traffic level on contracted farmland, and agricultural machinery.

#### 4.2.2. Model

According to the above theoretical analysis of the policy implementation–farmers’ input behavior in agricultural production–farmland transfer process, FRCP may affect farmland transfer through input in agricultural production. In this section, we further empirically examine the mechanism by which FRCP affects farmland transfers. This analysis is mainly based on the step-by-step method for sequential inspection, and the regression models are as follows:

(1) $$Y_{i}=c_{0}+c_{1}CERT_{i}+\sum c_{2}X_{i}+\mu_{1}$$

(2) $$M_{i}=a_{0}+a_{1}CERT_{i}+\sum a_{2}X_{i}+\mu_{2}$$

(3) $$Y_{i}=b_{0}+b_{1}CERT_{i}+b_{2}M_{i}+\mu_{3}$$

In Equations (1)–(3), *Y_i_* represents farmland transfer behavior (including farmland leasing in and leasing out), *M_i_* represents an intermediary variable (agricultural production incentive), *CERT_i_* represents FRCP, and *X_i_* represents control variables comprising household characteristics and contracted land characteristics. Equation (1) represents the total effect of FRCP on farmland transfer. Equation (2) represents the impact of land rights confirmation on the intermediary variable (agricultural production incentive). Equation (3) refers to the effect of FRCP on farmland transfer after the addition of intermediary variables. In terms of coefficient, *b*_2_ is the direct effect of the intermediary variable on farmland transfer. When combining Equations (2) and (3), the mediation effect can be obtained as *b*_2_*a*_1_—that is, the impact of FRCP on farmland transfer through the intermediary transmission mechanism.

We test for the mediation effect using the following steps: First, the coefficient *c*_1_ in Equation (1) is determined. If it has statistical significance, it is regarded as having a mediating effect. Otherwise, it is regarded as having a cover effect. Based on the result that the estimated coefficient *a*_1_ in Equation (1) has statistical significance, we then test whether FRCP affects the mediating variable (Equation (2)) and whether the mediating variable affects farmland transfer (Equation (3)) to a level of statistical significance. If both the estimated coefficients, *a*_1_ and *b*_2_, are statistically significant, it indicates that a mediation effect exists, which requires us to move to the fourth step. However, if at least one of the coefficients is not significant, then we need to perform a second test. Second, since the mediating effect model in this paper is nonlinear, the statistical significance of the null hypothesis *a*_1_ × *b*_2_ is tested using the bootstrap sampling method. If *a*_1_ × *b*_2_ is significant, indicating the existence of mediating effects, we should carry out the third step, or stop the analysis. Third, we test the coefficient *b*_1_ of Equation (3). If *b*_1_ is not statistically significant, a perfect mediation effect exists in the model. If *b*_1_ is statistically significant, the symbols of *a*_1_ × *b*_2_ and *b*_1_ are compared. If the symbols are consistent, it indicates that there is a partial mediation effect; otherwise, there is a cover effect. Finally, the mediating effects, i.e., *a*_1_ × *b*_2_*/c*_1_, or cover effects, *|a*_1_ × *b*_2_*/c*_1_*|*, are reported in the results.

## 5. Results

### 5.1. Empirical Test and Analysis

This research conducts regression analysis on the total effect of FRCP on farmland transfer behavior, including farmland leasing out and leasing in, using Stata 14.0 software and the Probit model. Table 2 shows the variance inflation factor (VIF) values before model estimation. The greatest VIF value for all variables was 1.67, the minimum was 1.15, and the mean was 1.32; because these values are all less than 2, there is no severe multicollinearity concern between variables in this article.

The results of the regression analysis of comprehensive effects are shown in Table 3. FRCP has a negative impact on land leasing out at a significance level of 5%, while it has a positive impact on land leasing in at a significance of 1%. Moreover, the longer the time period of confirmed farmland right, the more difficult it is for farmers to lease out their land. It is also indicated that FRCP may strengthen farmers’ endowment effect of farmland, and then restrain them from the behavior of farmland leasing out. On the contrary, if farmers believe that the duration of their land use rights can remain unchanged for a long time, the possibility for them to expand their production and operation scale will be increased.

### 5.2. Robustness Results

In this paper, the bootstrap sampling method was adopted, setting to put back the 1000 times. Robustness test results are shown in Table 4. The significance results, coefficient sizes, and sign directions are comparable to those of the original model, indicating that the empirical model tested in this paper is relatively robust.

### 5.3. Mechanism Analysis

Results on the mediating effect of the agricultural production input on farmland leasing out and leasing in are shown in Table 5 and Table 6, respectively. Accordingly, the coefficient value of path I represents the effect of FRCP on the intermediary mechanism, which corresponds to the value of coefficient *a*_1_ in Equation (2) in Section 5.2. In addition, the coefficient value of path II represents the effect of FRCP on the intermediary mechanism, which corresponds to the value of coefficient *b*_2_ in Equation (3). Moreover, the mediating effect values in Table 5 and Table 6 represent the influence of FRCP on farmland leasing out and leasing in behavior, respectively, through mediating variables, namely the coefficient *b*_2_*a*_1_ above. Meanwhile, the proportion of the mediating effect is shown in the last column of the following two tables.

First, FRCP promotes the input of agricultural capital and agricultural time at a significance of 5%, while input in agricultural capital and agricultural time has a negative effect on the leasing out of farmland. Overall, confirmation of farmland rights has a negative impact on leasing out of farmland through the two intermediary variables, agricultural capital input and agricultural time input. In other words, when the period for confirming farmland rights is extended, farmers’ involvement in agricultural output will expand as well. Farmers will not lease out their lands in order to increase their income for owner-cultivation. Accordingly, FRCP also, with a confidence interval of 5%, promotes the input of agricultural capital and agricultural time. However, the input of agricultural capital and agricultural time has a positive effect on the transfer in behavior, which is opposite to that of leasing out, that is to say, farmers will lease in farmland to expand the original production scale to earn more profits. These findings, respectively, verify Hypotheses 3 and 4. According to in-depth interviews, FRCP gives farmers a strong sense of entitlement that will become stronger over time, increasing their incentive to invest in land. Then, farmers see more positive and favorable factors in their agricultural output, such as cultivated land resources, as they accumulate agricultural capital and time input. Meanwhile, the accumulation of benefits in agricultural production further enhances their cognition of the benefits of farmland transfer behavior. Thus, in order to improve the total yield of production, farmers usually need to expand the scale of their production and operation through farmland leasing in [47]. Relatively, the possibility of farmland leasing out may be reduced.

Second, the mediating effect value of the input in agricultural capital and input in agricultural time are −0.100 and −0.089, respectively. In terms of farmland leasing in, the mediating effect value is 0.116 and 0.097, respectively. To sum up, whether leasing out or leasing in farmland, the mediating effect of input in agricultural capital is greater, accounting for 15.504% and 14.536%, respectively, followed by input in agricultural time, which only accounts for 13.798% and 12.155%, respectively. It can be seen that FRCP mainly restrains farmland leasing out but promotes farmland leasing in through the input of agricultural capital.

## 6. Discussion

This paper has contributed to our understanding of the FRCP from the perspective of farmers. As a matter of national governance, in the process of transition from a traditional agricultural society to a modern industrial society, the change of land property relations involves the interaction between different social groups, and farmers, as the end audience of policy, their feedback on the effect of policy implementation can most directly reflect the performance of the institutional design. On the one hand, we consider the effects of policy implementation on the lease-in and lease-out behavior of farm households, respectively, and on the other hand, we consider the realistic paths of action of agricultural production inputs. These can provide more detailed feedback for our understanding of the policy implementation in Jiangxi Province and similar regions. We applied the deep interview and questionnaire to present an analysis framework consisting of “policy implementation-input behavior in agricultural production -farmland transfer” and testify the framework with the mediation model.

This paper reveals the impact of FRCP on farmers’ transfer behavior from the perspective of farmers through the “post-evaluation” approach. Previous relevant studies cast an eye over a policy that is being implemented, which supports a great way to obtain feedback on the barriers to implementation timely and quickly. However, as a multi-subject of China’s rural land reform, it is clear that analyzing the “after-effects” of the implementation of FRCP, especially the feedback of farmers’ subjects on the policy, is a better way to capture the stable decision-making behavior of farmers. In our study, we picked “the duration of FRCP” rather than “whether or not to confirm farmland rights” as a key explanatory variable.

Data from the seventh national census shows that 18.7% of China’s total population is already over 60 years old. Meanwhile, China’s urbanization rate reaches 64.7% in 2021. This means that China may have a shortage of agricultural labor in the future, who will cultivate the land, especially the abandoned land, and how skills and funds can achieve a reasonable bidirectional flow between rural and urban areas have become difficult issues for government decision-makers [14]. Similar to existing studies, for example, France has constructed a series of laws and rules to solve the contradiction between fragmented farmland ownership and moderate-scale agricultural operation since the 1960s, and our paper also confirms that FRCP has a positive effect on enhancing agricultural land transfer behavior. By analyzing the mechanisms that influence farmland transfer behavior, we expand our knowledge of the pathway for the continued role of FRCP in the future. The implementation performance of FRCP may be influenced not only by the factor of agricultural production inputs but also by a combination of other economic and social factors.

There are some limitations in this study. Although we provide empirical evidence to better understand FRCP from a farmer’s perspective, more research is required to fully comprehend our findings. Furthermore, in our study, we picked “the duration of FRCP” as the key explanatory variable. The effective impact of the duration of confirmed farmland right is still difficult to describe scientifically, necessitating additional refining in future studies. A bigger sample size, on the other hand, will yield more reliable mathematical statistics results.

## 7. Conclusions

In general, FRCP has a significant negative impact on the behavior of leasing out farmland, while it has a significant positive impact on the behavior of leasing in farmland. Further, the mediating effect analysis shows that changes in farmland transfer behavior can be explained by improving input in agricultural production. Specifically, in the leasing out farmland model, the mediating effect of agricultural capital input and agricultural time input account for 15.504% and 14.536% of the total utility, respectively. In the leasing in farmland model, these two mediating effects accordingly account for 13.798% and 12.155% of the total effects. At the same time, farmers have progressively developed a strong sense of entitlement as a result of the legislation of FRCP and its countrywide implementation. As time goes on, this sense of entitlement will grow, which has led farmers to become more motivated in farming.

Several policy implications may be deduced from the preceding results and the validated mechanism. First, it is critical to actively investigate new types of rights, such as mortgages, loans, and credit systems for farmers. Second, the government should invest more in rural mechanized production based on regional realities, modernize the rural transportation system to fulfill the requirements for mechanized agriculture, and give farmers production incentives. Third, in hilly mountainous areas where smallholder farming is the mainstay, fostering market supply agents for agricultural production factors can effectively reduce farmers’ transaction costs, promoting the formation of a modern agricultural industry system. Fourth, although FRCP has considerably decreased the transaction barrier in the farmland transfer process, it is still necessary to consider how to coordinate all aspects of the policy implementation process. Many measures such as family farm policy, differentiation subsidy policy, and so on are helpful for FRCP in practice.

## Figures and Tables

**Table 1:** The definition of the variables.

Variables		Definitions
Explained variables	Transfer leasing out	Whether to lease out (1 = yes, 0 = no)
	Transfer leasing in	Whether to lease in (1 = yes, 0 = no)
Explanatory variable	Farmland rights confirmation policy (FRCP)	Difference between the year of survey and the year in which FRCP certificate was issued (year)
Mediating variables	Agricultural input	Agricultural capital input; namely, total annual capital input for agricultural production (yuan)
		Agricultural time input—i.e., total amount of annual time invested in agricultural production (hours)
Control variables	Age	Age of household head (years)
	Education level	1 = primary school or below; 2 = junior middle school; 3 = technical secondary school or high school; 4 = college degree or above
	Official experience	Whether the head of the household has served as a village head (1 = yes, 0 = no)
	Nonfarmer size	Proportion of nonagricultural labor force in the family
	Agricultural machinery	Whether the household owns agricultural machinery (1 = yes, 0 = no)
	Contracted farmland area	Families’ contracted land area in the second round of family contracting
	Traffic level on contracted land	1 = low; 2 = medium; 3 = high
	Contracted farmland reallocation	Whether the farmland was adjusted during the second round of family contracting (1 = yes, 0 = no)

**Table 2:** VIF value test results of independent variables.

Variable	VIF Value
FRCP	1.13
Age	1.65
Education level	1.23
Village cadre experience	1.27
Nonfarmer size	1.14
Contracted farmland reallocation	1.13
Agricultural machinery	1.53
Contracted farmland area	1.12
Traffic level on contracted land	1.13
Mean of the VIF	1.26

**Table 3:** Estimation results of the impact of FRCP on farmland transfer behavior.

Variable	Farmland Leasing Out	Farmland Leasing In
FRCP	−0.645 ** (0.184)	0.798 *** (0.209)
_cons	0.218 (0.685)	0.356 (0.534)
Prob > chi^2^	0.000	0.000
LR chi^2^	50.871	55.794
Pseudo R^2^	0.278	0.398

Note: Due to space limitations, the estimated results of other control variables are omitted. ***: significant at the 1 percent level; **: significant at the 5 percent level;.Standard errors are in parentheses.

**Table 4:** Robustness check results of the benchmark model.

	Bootstrap	
	Farmland Leasing Out	Farmland Leasing In
FRCP	−0.595 ** (0.211)	0.737 *** (0.245)
Control variables	Controlled	Controlled
Constant	0.253 (0.773)	0.227 (0.698)
Pseudo R^2^	0.047	0.046

Note: ***: significant at the 1 percent level; **: significant at the 5 percent level; Standard errors are in parentheses.

**Table 5:** The impact of FRCP on farmland leasing out: an analysis of the mediating effect.

Path I Influence of FRCP on the Intermediary Mechanism	Coefficient	Path II Influence of the Intermediary Mechanism on Farmland Leasing Out	Coefficient	Mediating Effect Value	Proportion of Mediating Effect (%)
The influence of FRCP on agricultural capital input	0.362 ** (0.290)	The influence of Agricultural capital input on farmland leasing out	−0.278 ** (0.213)	−0.100 ** (0.195)	15.504
The influence of FRCP on agricultural time input	0.387 ** (0.322)	The influence of Agricultural capital input on farmland leasing out	−0.235 ** (0.226)	−0.089 * (0.092)	13.798

Note: **: significant at the 5 percent level; *: significant at the 10 percent level. Standard errors are in parentheses.

**Table 6:** The impact of FRCP on the transfer in of farmland: an analysis of the mediating effect.

Path I Influence of FRCP on the Intermediary Mechanism	Coefficient	Path II Influence of the Intermediary Mechanism on Farmland Leasing In	Coefficient	Mediating Effect Value	Proportion of Mediating Effect (%)
The influence of FRCP on agricultural capital input	0.384 ** (0.245)	The influence of Agricultural capital input on farmland leasing in	0.302 ** (0.296)	0.116 ** (0.130)	14.536
The influence of FRCP on agricultural time input	0.432 *** (0.389)	The influence of Agricultural time input on farmland leasing in	0.224 * (0.104)	0.097 * (0.046)	12.155

Note: *** *p* < 0.01, ** *p* < 0.05, and * *p* < 0.1 Standard errors are in parentheses.

## Data Availability

The data comes from questionnaires of farmers and they are not publicly available due to privacy restrictions. The data presented in this study are available on request from the corresponding author.
